# The Impact of the Triglyceride-Glucose Index on Poor Prognosis in NonDiabetic Patients Undergoing Percutaneous Coronary Intervention

**DOI:** 10.3389/fendo.2021.710240

**Published:** 2021-08-19

**Authors:** Jie Yang, Yi-Da Tang, Yitian Zheng, Chen Li, Qing Zhou, Jun Gao, Xiangbin Meng, Kuo Zhang, Wenyao Wang, Chunli Shao

**Affiliations:** ^1^Department of Cardiology, State Key Laboratory of Cardiovascular Disease, Fuwai Hospital, National Center for Cardiovascular Diseases, Chinese Academy of Medical Sciences and Peking Union Medical College, Beijing, China; ^2^Department of Cardiology, Institute of Vascular Medicine, Peking University Third Hospital, Key Laboratory of Molecular Cardiovascular Science, Ministry of Education, Beijing, China; ^3^Department of Cardiology, Zhengzhou University People’s Hospital, Zhengzhou, China; ^4^Department of Cardiology, Central China Fuwai Hospital, Central China Branch of the National Cardiovascular Center, Zhengzhou, China; ^5^Department of Cardiology, Henan Provincial People’s Hospital, Zhengzhou, China

**Keywords:** TyG index, CAD, PCI, MACCE, non-diabetic

## Abstract

**Background:**

The triglyceride-glucose index (TyG index) is a valuable marker for predicting adverse cardiovascular events in diabetic patients. However, for nondiabetic patients, whether the TyG index is independently related to poor prognosis remains unclear. This cohort study assessed the association of the TyG index with future cardiovascular risk in nondiabetic subjects who received percutaneous coronary intervention (PCI).

**Methods:**

We consecutively enrolled 5,489 nondiabetic patients who underwent PCI. All experimental subjects were divided into three groups based on their TyG index, which was determined by the equation ln (fasting triglyceride (mg/dl) × fasting blood glucose (mg/dl)/2). The primary endpoint was major adverse cardiovascular and cerebrovascular events (MACCE), including all-cause death, nonfatal myocardial infarction (MI), nonfatal stroke, and target vessel revascularization (TVR).

**Results:**

A total of 386 MACCE were documented during a median 29-month follow-up. The Kaplan–Meier survival results indicated that among the three groups, there was no obvious difference in any endpoints. Further Cox regression analyses suggested that the TyG index was not independently related to adverse cardiovascular outcomes for nondiabetic patients who underwent PCI (HR: 0.77, 95% CI 0.56–1.16, *P* = 0.210 for MACCE). Subgroup analysis suggested that the TyG index was independently relevant to MACCE for patients with low-density lipoprotein cholesterol (LDL-C) lower than 1.8 mmol/L.

**Conclusion:**

The TyG index is not an effective predictive factor for adverse cardiovascular prognosis in nondiabetic patients who underwent PCI. However, in subjects with LDL-C lower than 1.8mmol/L, it may predict future cardiovascular risk.

## Introduction

Cardiovascular and cerebrovascular diseases, particularly coronary artery disease (CAD), are the main cause of death (1), which leads to a large social and economic burden. With the improvement and popularization of standardized primary prevention and secondary prevention strategies for CAD, the incidence of adverse outcomes in CAD patients has been greatly reduced (2, 3). However, with the improvement of living standards, the incidence of metabolic abnormalities such as insulin resistance (IR), metabolic syndrome (MS), hyperuricemia, and nonalcoholic fatty liver disease (NAFLD) has increased remarkably (4, 5). Metabolic abnormalities with insulin resistance as the core are important risk factors for arteriosclerotic cardiovascular diseases (ASCVDs) and cardiovascular adverse outcomes, especially coronary heart disease (6–9).

The direct measure of IR is the hyperinsulinemic euglycemic glucose clamp. However, as a result of its complexity and invasiveness, it is rarely applied in clinical work. Homeostasis model assessment of IR (HOMA-IR) is commonly used clinically to assess IR, and some recent research has indicated that the TyG index is also a credible indicator of IR (10–12). Many researchers have previously explored the relevance between this index and poor prognosis in patients with diabetes mellitus (DM) or acute coronary syndrome (ACS), especially in patients with myocardial infarction (13–16), but few studies have focused on its predictive value for poor outcomes in nondiabetic patients who received percutaneous coronary intervention (PCI). Therefore, we carried out this study to assess the correlation between the TyG index and adverse cardiovascular prognosis in nondiabetic participants who accepted PCI treatment.

## Methods

### Study Design and Participants

This research is a prospective observational cohort study that conformed to the Declaration of Helsinki and was authorized by the Fuwai Hospital’s Ethics Review Committee. All participants signed informed consent before PCI.

**Figure 1:**
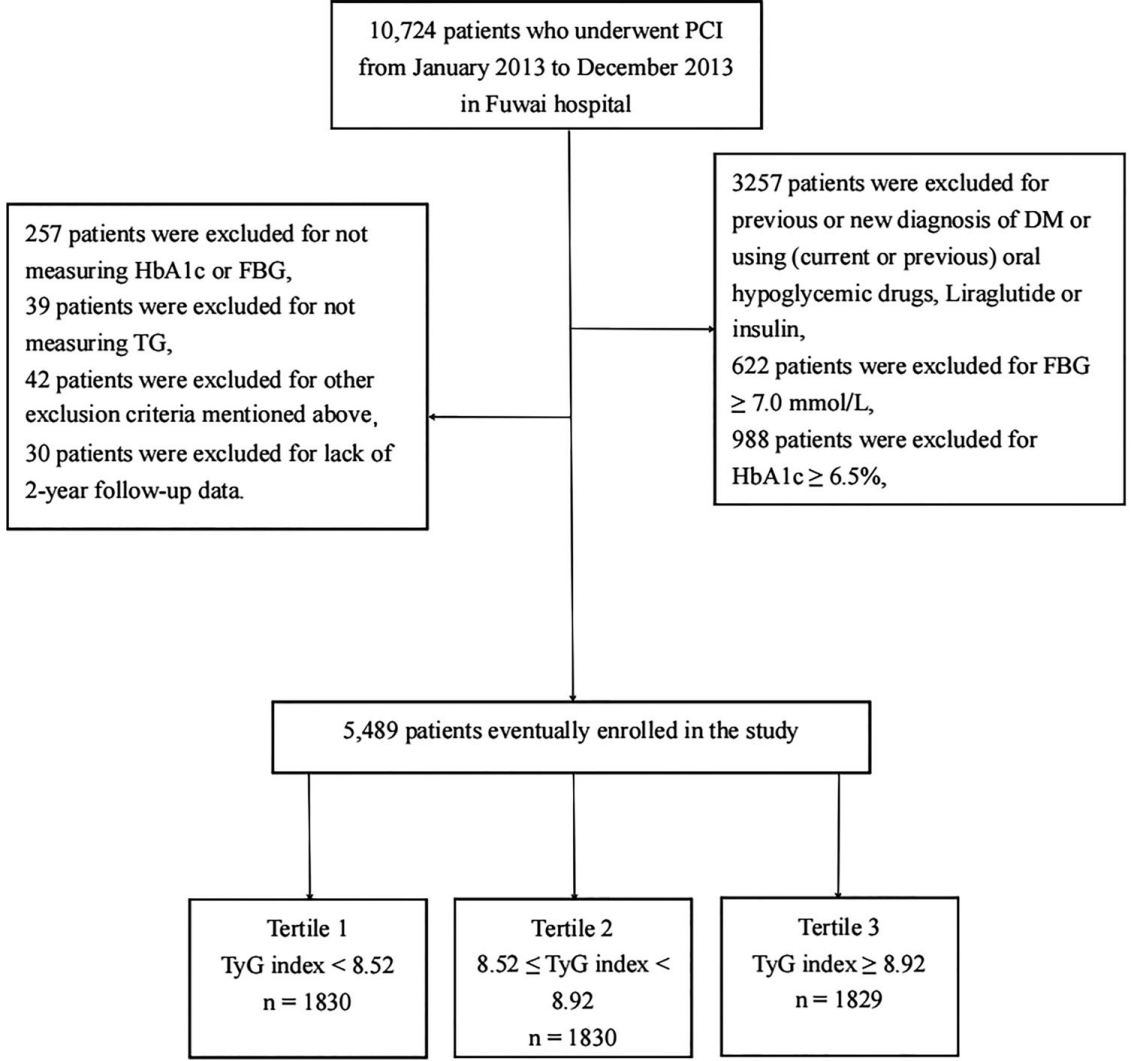
Flowchart of the study population enrollment. PCI, percuntaneous coronary intervention; DM, diabetes mellitus; FBG, fasting blood glucose; HbA1c, Hemoglobin A1c; TG, triglycerides, TyG index, triglyceride-glucose index.

We consecutively enrolled 10,724 patients with CAD who had received PCI in Fuwai Hospital, Chinese National Center for Cardiovascular Diseases, from January 2013 to December 2013. According to the exclusion criteria, 3,257 patients were excluded for previous or new diagnosis DM or using (current or previous) oral hypoglycemic drugs, liraglutide or insulin; 1,610 patients were excluded for hemoglobin A1c (HbA1c) ≥6.5% or fasting blood glucose (FBG) ≥7.0 mmol/L; and 296 participants were excluded for not measuring HbA1c, FBG, or triglyceride (TG). Patients with very old age (age > 80 years), cardiogenic shock, severe renal insufficiency with endogenous creatinine clearance rate <30 ml/min/m^2^, extreme obesity [baseline body mass index (BMI) > 45 kg/m^2^], or lack of 2-year follow-up data were also excluded, and the detailed population screening process is shown in **Figure 1**. Finally, we enrolled 5,489 nondiabetic patients who underwent PCI. All participants were divided into three groups based on their admission tertiles of the TyG index: group 1 (TyG index < 8.52, *n* = 1,830), group 2 (8.52 ≤ TyG index < 8.92, *n* = 1,830), and group 3 (TyG index ≥ 8.92, *n* = 1,829).

### Data Measurement and Definitions

We collected baseline data consisting of age, sex, BMI, smoking history, types of CAD, previous history of hypertension, hyperlipemia, myocardial infarction (MI), hemorrhagic or ischemic stroke, PCI, and coronary artery bypass graft (CABG). Imaging and laboratory examinations, including left ventricle ejection fraction (LVEF), lipid parameters [total cholesterol (TC), high-density lipoprotein cholesterol (HDL-C), low-density lipoprotein cholesterol (LDL-C), and TG], glycemic parameters (HbA1c and FBG), high-sensitivity C-reactive protein (hs-CRP), and creatinine, were also measured by standardized methods, and laboratory tests were performed after an overnight fast. FBG, TG, TC, HDL-C, LDL-C, hs-CRP, and creatinine were assayed using a LABOSPECT 008 system (Hitachi, Tokyo, Japan), and the HbA1c value was determined by high-performance liquid chromatography (G8, TOSOH, Tokyo, Japan).

After coronary angiography and PCI were completed, characteristics of coronary stenosis [number of narrow coronary vessels, special types of CAD, and Synergy between PCI with TAXUS™ and cardiac surgery (SYNTAX) score] and the diameter and number of stents were evaluated by two coronary intervention experts who did not know the baseline data of the subjects before the evaluation (17). BMI index can be computed based on the following formula: BMI = weight (kg)/height (m^2^). Hyperlipemia was defined as a past diagnosis with hyperlipemia or elevated blood lipids on admission. Hypertension was defined as a previous diagnosis with hypertension or using oral antihypertension drugs previously or currently. Stroke was defined as a previous diagnosis of ischemic cerebral stroke, transient ischemic attack (TIA), or cerebral hemorrhage. In addition, we classified the types of CAD, including chronic coronary syndrome (CCS), unstable angina (UA), and acute myocardial infarction (AMI), according to the relevant guidelines (18–20). The TyG level was computed by the following equation: ln (fasting triglyceride (mg/dl) × fasting glucose (mg/dl)/2) (21). LM disease was defined as left main coronary artery stenosis ≥50%. Multivessel disease can be determined according to ≥2 epicardial coronary artery stenoses over 50% located in different vascular systems [left anterior descending branch (LAD), left circumflex branch (LCX), and right coronary artery (RCA)]. We defined chronic total occlusion (CTO) disease as the lumen of the coronary artery being completely occluded [thrombolysis in myocardial infarction (TIMI) flow grade 0 or 1] for more than 3 months (22). In-stent restenosis (ISR) disease was defined as stenosis greater than 50% in the stent or within 5 mm at both ends of the stent (23). The diameter of the stent is defined as the diameter of the smallest stent to be implanted.

### Follow-Up and Endpoint Definition

We followed up with the patients at 1, 6, 12, and 24 months by telephone, letter, and clinic visits. All of the follow-up personnel were trained strictly and did not know the baseline data of patients. The primary endpoint was major adverse cardiovascular and cerebrovascular events (MACCE), including all-cause mortality, nonfatal MI, nonfatal stroke, and target vessel revascularization (TVR). The secondary outcomes included MACE (a composite of cardiac death, nonfatal MI, and TVR), all-cause death, nonfatal MI, nonfatal stroke, and TVR. All-cause mortality was defined as any reason of death. We defined cardiac death as death from sudden death, heart failure, myocardial infarction, fatal arrhythmia, or any other fatal cardiovascular disease. Stroke and MI were defined as mentioned above. Target vessel revascularization was defined as ischemia-driven or clinically driven targeted vessel interventional therapy or surgery. We continued to follow the patients for up to 2 years to achieve the primary endpoint unless the patients died or were lost.

### Statistical Analyses

All statistical analyses and figures were treated by SPSS (version 23, Chicago, IL, USA) and R language version 4.0.3. If the continuous variables conformed to a normal distribution, they were described by the mean plus or minus the standard deviation, while other variables that were not in accordance with a normal distribution were shown as the median (interquartile range). Categorical variables are represented as quantities and percentages. We used ANOVA to compare the differences in measurement data among the three groups. For continuous variables that did not coincide with a normal distribution, the rank-sum test was applied to assess the differences between the three groups. We used the chi-square test to compare the categorical variables. Log-rank tests and K-M survival analyses were used to explore differences in long-term survival under different endpoint definitions. We performed collinearity analysis to explore variables that were significantly associated with the TyG index. Independent predictive factors for adverse cardiovascular events were determined by the Cox regression method. In the multivariate regression analysis, we included both traditional cardiovascular risk factors and variables that were intimate relative to prognosis in the univariate regression process. Variables that were significantly collinear with the TyG index were excluded. In addition, we further explored the effect of the TyG index on future cardiovascular events in different subgroups, including obesity, abnormal glucose metabolism, old age, and AMI. A two-sided *P* < 0.05 was set as the statistical threshold for all tests.

## Results

### Baseline Characteristics

A total of 5,489 nondiabetic subjects were selected for the present study. Their average age was 57.20 ± 10.22 years old, 4,358 (79.4%) patients were male, overweight or obese patients accounted for 22.3%, 3,324 (60.6%) patients had a hypertension history, 3,482 (63.4%) patients were diagnosed with hyperlipemia, 3,221 (58.7%) subjects were current smokers, and 1,354 (24.7%) subjects had previously undergone PCI or CABG. All enrolled subjects were separated into three groups according to baseline TyG level [tertile 1 (*n* = 1,830): TyG index ≤ 8.52; tertile 2 (*n* = 1,830): 8.52 ≤ TyG index ≤ 8.92; tertile 3 (*n* = 1,829): TyG index ≥ 8.92]. Detailed baseline data are presented in **Table 1**. We found that there were significant differences among the three groups in terms of age, BMI, history of hyperlipemia, history of stroke, smoking history, type of CAD, percentage of diffuse and CTO disease, HbA1c, FBG, TC, TG, LDL-C, HDL-C, hs-CRP, and creatinine. There was no significant difference in other baseline characteristics, including sex, history of hypertension, previous MI, previous PCI, or CABG. To explore baseline characteristics closely related to the triglyceride-glucose index, collinearity analysis was performed. We found that TC and LDL-C had a high correlation with the TyG index, for which the VIFs were all more than 10. In addition, there was some correlation between TG and the TyG index, for which the VIF was 4.893. Detailed collinear diagnosis results are shown in **Table 2**.

**Table 1:** Baseline demographic and clinical data of the three groups.

Variable	Tertile 1 (*n* = 1,830)	Tertile 2 (*n* = 1,830)	Tertile 3 (*n* = 1,829)	*P*-value
TyG index	8.24 ± 0.21	8.72 ± 0.11	9.26 ± 0.30	<0.001
Age, years	59.14 ± 10.20	57.07 ± 10.30	55.10 ± 9.82	<0.001
Male, *n* (%)	1,467 (80.2)	1,449 (79.2)	1,442 (78.8)	0.341
BMI, kg/m^2^	24.84 ± 3.32	25.73 ± 3.02	26.39 ± 3.01	<0.001
Hypertension, *n* (%)	1,087 (59.4)	1,100 (60.1)	1,137 (62.2)	0.084
Hyperlipemia, *n* (%)	1,038 (56.7)	1,166 (63.7)	1,278 (70.4)	<0.001
Current smoker, *n* (%)	1,038 (56.7)	1,052 (57.5)	1,131 (61.8)	0.003
Previous stroke, *n* (%)	171 (9.3)	194 (10.6)	125 (6.8)	0.007
Previous MI, *n* (%)	500 (27.3)	544 (29.7)	519 (28.4)	0.489
Previous PCI, *n* (%)	413 (22.6)	362 (19.8)	378 (20.9)	0.147
Previous CABG, *n* (%)	71 (3.9)	68 (3.7)	62 (3.4)	0.446
Types of CAD				
CCS, *n* (%)	498 (27.2)	564 (30.8)	522 (28.5)	
UA, *n* (%)	1,418 (59.3)	1,365 (54.0)	1,337 (54.1)	0.036
AMI, *n* (%)	246 (13.4)	277 (15.1)	318 (17.4)	
LM disease, *n* (%)	102 (5.6)	107 (5.8)	91 (5.0)	0.468
Multivessel disease, *n* (%)	1,286 (70.3)	1,309 (71.5)	1,331 (72.7)	0.098
Diffuse disease, *n* (%)	1,030 (56.3)	1,076 (58.8)	1,129 (61.7)	0.001
CTO disease, *n* (%)	140 (7.7)	144 (7.9)	175 (9.6)	0.034
ISR disease, *n* (%)	124 (6.8)	127 (6.9)	113 (6.2)	0.498
SYNTAX score	9 (6, 16)*	10 (6, 16)*	10 (6, 16)*	0.276
Diameter of stent	2.75 (2.5, 3.0)*	2.75 (2.5, 3.0)*	2.75 (2.5, 3.0)*	0.614
Numbers of stent	2 (1, 2)*	2 (1, 2)*	2 (1, 2)*	0.126
LVEF, (%)	63.87 ± 6.74	63.09 ± 7.15	63.15 ± 7.21	0.005
FBG, mmol/L	4.96 ± 0.53	5.16 ± 0.56	5.34 ± 0.61	<0.001
HbA1c (%)	5.85 ± 0.34	5.88 ± 0.33	5.91 ± 0.33	<0.001
TG, mmol/L	0.99 ± 0.20	1.51 ± 0.23	2.63 ± 1.23	<0.001
TC, mmol/L	3.78 ± 0.93	4.19 ± 1.00	4.67 ± 1.07	<0.001
HDL-C, mmol/L	1.13 ± 0.30	1.05 ± 0.28	0.97 ± 0.24	<0.001
LDL-C, mmol/L	2.24 ± 0.81	2.52 ± 0.89	2.76 ± 0.95	<0.001
hs-CRP, mg/L	1.11 (0.53, 2.62)*	1.46 (0.76, 3.21)*	1.75 (0.97, 3.71)*	<0.001
Creatinine, mmol/L	73.94 ± 13.54	75.28 ± 14.44	75.89 ± 14.65	0.001

Data are represented as mean ± SD, medians with interquartile ranges* or n (%).

TyG index, triglyceride-glucose index; BMI, body mass index; MI, myocardial infarction; PCI, percutaneous coronary intervention; CABG, coronary artery bypass graft; CAD, coronary artery disease; CCS, chronic coronary syndrome; UA, unstable angina; LM, left main; CTO, chronic total occlusion; ISR, in-stent restenosis; SYNTAX, Synergy between PCI with TAXUS™ and cardiac surgery; LVEF, left ventricle ejection fraction; FBG, fasting blood glucose; HbA1c, Hemoglobin A1c; TG, triglycerides; TC, total cholesterol; HDL-C, high-density lipoprotein cholesterol; LDL-C, low-density lipoprotein cholesterol; hs-CRP, high-sensitivity C-reactive protein.

**Table 2:** Collinearity analysis of baseline data with TyG index.

	Unstandardized coefficients		Standardized coefficients	*t*	Sig.	Collinearity statistics	
	*B*	Std. error	Beta			Tolerance	VIF
(Constant)	6.683	0.101		66.228	0.000		
Age	−0.001	0.000	−0.015	−1.695	0.090	0.768	1.303
Sex	0.055	0.013	0.045	4.328	0.000	0.569	1.758
BMI	0.003	0.001	0.023	2.649	0.008	0.861	1.162
Hypertension	−0.003	0.008	−0.003	−0.342	0.733	0.904	1.106
Hyperlipemia	0.023	0.008	0.023	2.844	0.005	0.937	1.067
Previous stroke	−0.013	0.013	−0.008	−0.980	0.327	0.952	1.050
Current smoker	0.008	0.009	0.009	0.929	0.353	0.746	1.340
Previous MI	0.024	0.009	0.023	2.844	0.005	0.937	1.067
Previous PCI	−0.014	0.012	−0.011	−1.100	0.271	0.664	1.507
Previous CABG	0.015	0.029	0.005	0.530	0.596	0.871	1.148
AMI	−0.009	0.010	−0.007	−0.832	0.406	0.940	1.064
LM disease	0.001	0.012	0.000	0.072	0.942	0.877	1.140
Multivessel disease	−0.001	0.010	−0.001	−0.146	0.884	0.895	1.118
Diffuse disease	0.008	0.011	0.006	0.679	0.497	0.887	1.128
CTO disease	0.007	0.012	0.005	0.598	0.550	0.908	1.101
ISR disease	0.030	0.020	0.014	1.482	0.139	0.745	1.342
SYNTAX score	0.000	0.001	−0.003	−0.352	0.725	0.747	1.338
Diameter of stent	−0.001	0.009	−0.001	−0.122	0.903	0.924	1.083
Number of stent	0.001	0.006	0.001	0.113	0.910	0.918	1.089
LVEF	0.001	0.001	0.010	1.128	0.259	0.814	1.229
FBG	0.198	0.007	0.239	28.836	0.000	0.913	1.095
HbA1c	0.026	0.012	0.018	2.193	0.028	0.899	1.113
TG	0.482	0.009	0.956	54.579	0.000	0.204	4.893
TC	−0.184	0.024	−0.413	−7.714	0.000	0.022	45.680
HDL-C	0.014	0.026	0.008	0.529	0.597	0.290	3.448
LDL-C	0.232	0.025	0.444	9.399	0.000	0.028	35.563
hs-CRP	0.000	0.001	−0.003	−0.351	0.725	0.860	1.113
Creatinine	0.001	0.000	0.023	2.599	0.009	0.776	1.289

BMI, body mass index; MI, myocardial infarction; PCI, percutaneous coronary intervention; CABG, coronary artery bypass graft; AMI, acute myocardial infarction; LM, left main; CTO, chronic total occlusion; ISR, in-stent restenosis; SYNTAX, Synergy between PCI with TAXUS™ and cardiac surgery; LVEF, left ventricle ejection fraction; FBG, fasting blood glucose; HbA1c, hemoglobin A1c; TG, triglycerides; TC, total cholesterol; HDL-C, high-density lipoprotein cholesterol; LDL-C, low-density lipoprotein cholesterol; hs-CRP, high-sensitivity C-reactive protein.

### Clinical Outcomes for Adverse Cardiovascular Events

**Figure 2:**
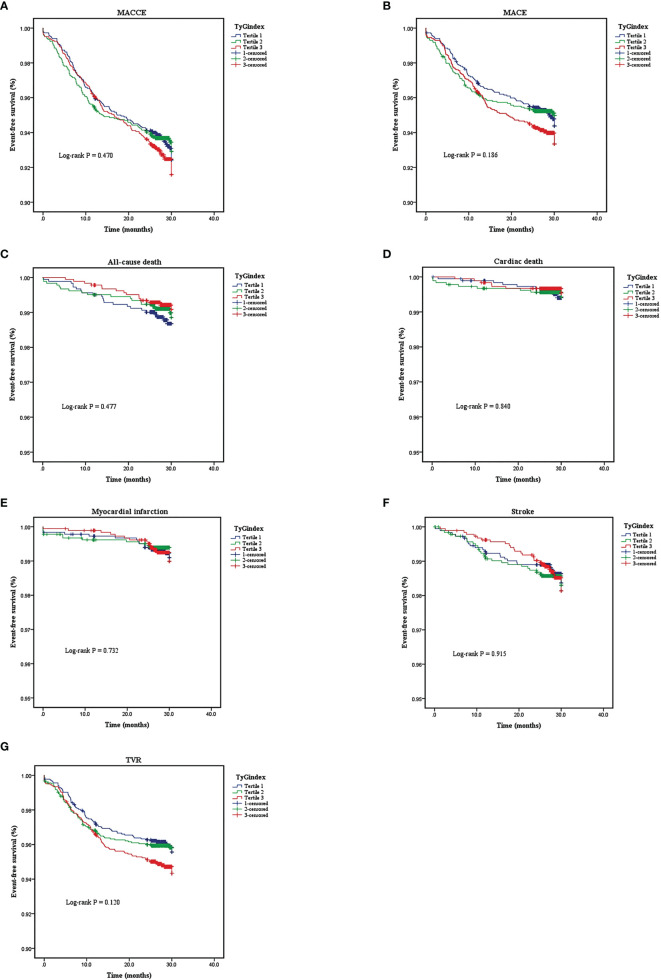
Kaplan-Meier analyses for different endpoints among the three groups: **(A)** major adverse cardiovascular or cerebrovascular events (MACCE), **(B)** major advances cardiovascular events (MACE), **(C)** all-cause death, **(D)** cardiac death, **(E)** myocardial infarction, **(F)** stroke, **(G)** target vessel revascularization (TVR).

**Figure 3:**
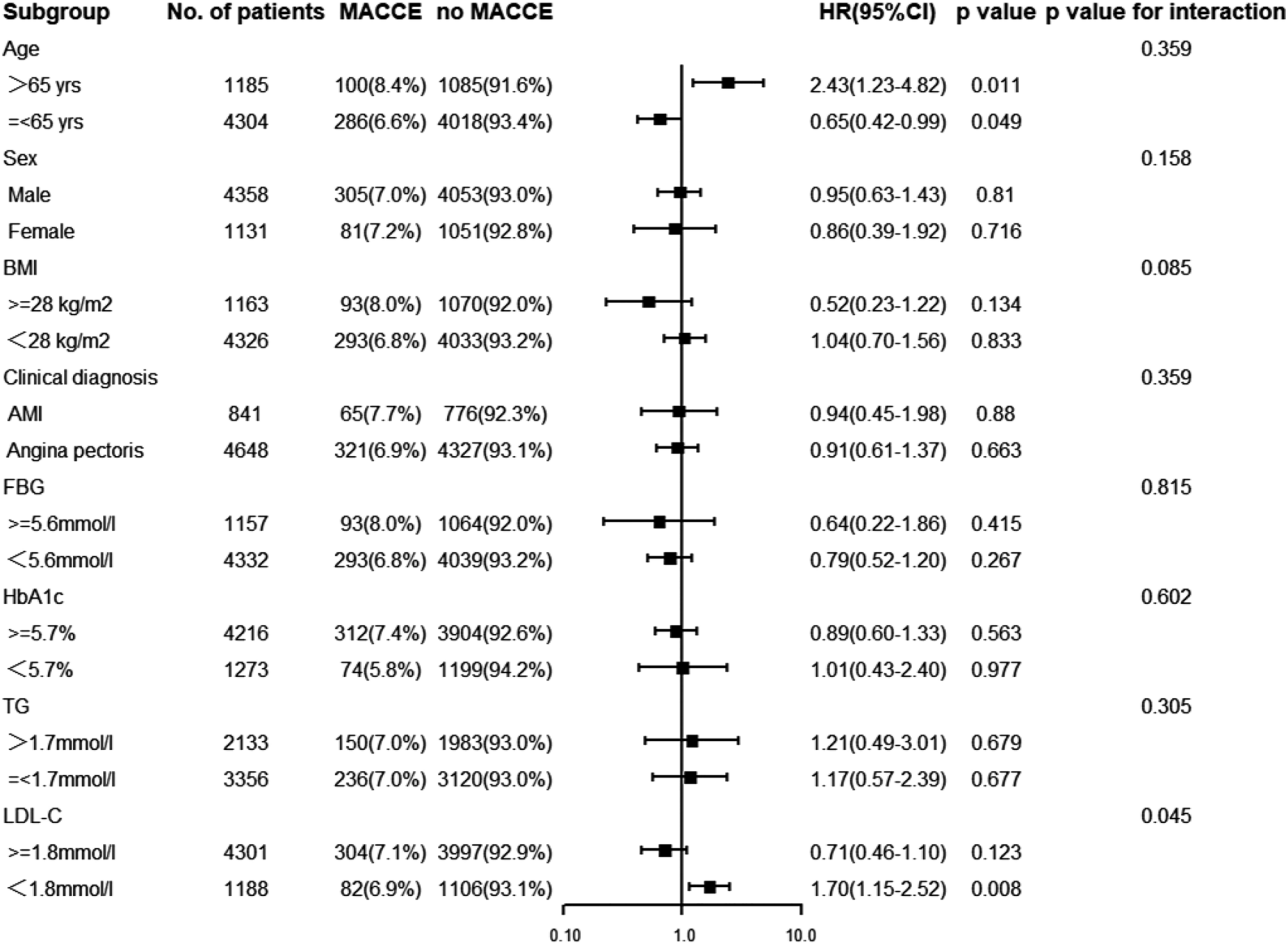
Subgroup analysis for the effect of the TyG index on the risk of the primary endpoint. The analysis was performed by adjusting for Model (age, gender, dyslipidemia, previous history of stroke, MI, PCI, and CABG, Multivessel disease, CTO disease, ISR disease, SYNTAX score, numbers of stent, LVEF, FBG, HbA1c and hs-CRP). BMI, body mass index; AMI, acute myocardial infarction; FBG, fasting blood glucose; HbA1c, glycosylated hemoglobin A1c; TG, triglyceride; LDL-C, low-density lipoprotein cholesterol; HR, hazard ratio; CI, confidence interval.

A total of 5,489 patients completed 2 years of follow-up, and the mean value was 29.0 months. A total of 386 MACCE; 296 MACE; 55 all-cause deaths, 25 of which were cardiac deaths; 40 nonfatal MIs; 81 nonfatal strokes; and 248 TVRs were documented. The Kaplan–Meier survival analyses indicated that there was no statistical discrepancy among the three groups regarding all endpoints (*P* = 0.470 for MACCE, *P* = 0.186 for MACE). Detailed outcomes of Kaplan–Meier survival analyses are presented in **Figure 2**. Then, Cox regression analyses were implemented to explore the independent risk factors for MACCE and MACE. Univariate Cox regression analyses showed that age, previous PCI, previous stroke, multivessel disease, CTO disease, ISR disease, SYNTAX score, number of stents, LVEF, FBG, HbA1c, and hs-CRP correlated with MACCE and that the TyG index, previous PCI, multivessel disease, CTO disease, ISR disease, SYNTAX score, diameter of stent, LVEF, HbA1c, and TG were related to MACE. TC and LDL-C were excluded from multivariate Cox regression models according to the outcomes of collinearity analysis. In multivariate analyses, we adjusted conventional risk factors and potential hazard factors that were notably associated with cardiovascular events in the univariate analyses (*P* < 0.1) and discovered that previous stroke, SYNTAX score, and LVEF were independently related to MACCE instead of the TyG index (HR: 0.77, 95% CI 0.56–1.16, *P* = 0.210) and that the diameter of the stent and SYNTAX score correlated with MACE independently rather than the TyG index (HR: 0.79, 95% CI 0.29–2.15, *P* = 0.650). Detailed outcomes of Cox regression analyses are shown in **Tables 3** and **4**. Subgroup analyses were completed to further assess the influence of the TyG level on adverse cardiovascular prognosis in different populations according to age (>65 or ≤65 years), sex (male or female), BMI (≥28 or <28 kg/m^2^), admission diagnosis (AMI or angina pectoris), FBG (≥5.6 or <5.6 mmol/L), HbA1c (≥5.7% or <5.7%), TGs (>1.7 or ≤1.7 mmol/L), and LDL-C (≥1.8 or <1.8 mmol/L). We discovered that the TyG level was an independent predictive factor for MACCE in the lower LDL-C subgroup (LDL-C < 1.8 mmol/L) but not in the higher LDL-C subgroup [HR (95% CI), 1.70 (1.15–2.52) for LDL-C < 1.8 mmol/L, *P* = 0.008 *vs*. 0.71 (0.46–1.10) for LDL-C ≥ 1.8 mmol/L, *P* = 0.123; *P* for interaction = 0.045]. The specific subgroup analysis results are illustrated in **Figure 3**.

**Table 3:** Univariate and multivariate Cox regression analyses for predicting the occurrence of MACCE.

Variable	Univariate analysis			Multivariate analysis		
	HR	95% CI	*P*-value	HR	95% CI	*P*-value
TyG index	1.16	0.95–1.44	0.148	0.77	0.52–1.16	0.210
Age, years	1.01	1.00–1.02	0.006	1.00	0.98–1.02	0.997
Male	1.03	0.81–1.32	0.815	0.93	0.58–1.48	0.925
BMI, kg/m^2^	1.01	0.98–1.04	0.537			
Hypertension	1.08	0.88–1.33	0.462			
Hyperlipemia	1.20	0.97–1.49	0.088	1.24	0.85–1.80	0.262
Current smoker	0.95	0.78–1.16	0.623			
Previous stroke	1.58	1.18–2.12	0.002	2.07	1.30–3.29	0.002
Previous MI	1.21	0.98–1.50	0.081	0.89	0.60–1.34	0.588
Previous PCI	1.46	1.17–1.82	0.001	1.31	0.79–2.18	0.291
Previous CABG	1.48	0.94–2.32	0.087	0.66	0.16–2.82	0.575
AMI	1.12	0.86–1.46	0.401			
LM disease	1.00	0.64–1.55	0.995			
Multivessel disease	1.44	1.13–1.84	0.003	1.50	0.90–2.50	0.122
CTO disease	2.00	1.51–2.65	<0.001	0.92	0.55–1.54	0.742
ISR disease	1.68	1.21–2.32	0.002	1.30	0.60–2.81	0.503
SYNTAX score	1.40	1.09–1.61	0.017	1.37	1.01–1.59	0.043
Diameter of stent	0.97	0.87–1.08	0.585			
Number of stents	1.03	1.02–1.04	<0.001	1.02	1.00–1.04	0.057
LVEF, (%)	0.97	0.96–0.99	<0.001	0.97	0.95–0.99	0.012
FBG, mmol/L	1.24	1.05–1.46	0.012	1.11	0.82–1.52	0.498
HbA1c (%)	1.51	1.10–2.06	0.010	1.62	0.91–2.89	0.102
TG, mmol/L	1.08	0.98–1.19	0.103			
TC, mmol/L	1.03	0.94–1.13	0.533			
HDL-C, mmol/L	0.83	0.58–1.19	0.312			
LDL-C, mmol/L	1.03	0.92–1.14	0.649			
hs-CRP, mg/L	1.03	1.01–1.06	0.009	1.01	0.97–1.06	0.210
Creatinine, mmol/L	1.00	1.00–1.01	0.346			

TyG index, triglyceride-glucose index; BMI, body mass index; MI, myocardial infarction; PCI, percutaneous coronary intervention; CABG, coronary artery bypass graft; CAD, coronary artery disease; AMI, acute myocardial infarction; LM, left main; CTO, chronic total occlusion; ISR, in-stent restenosis; SYNTAX, Synergy between PCI with TAXUS™ and cardiac surgery; LVEF, left ventricle ejection fraction; FBG, fasting blood glucose; HbA1c, hemoglobin A1c; TG, triglycerides; TC, total cholesterol; HDL-C, high-density lipoprotein cholesterol; LDL-C, low-density lipoprotein cholesterol; hs-CRP, high-sensitivity C-reactive protein.

**Table 4:** Univariate and multivariate Cox regression analyses for predicting the occurrence of MACE.

Variable	Univariate analysis			Multivariate analysis		
	HR	95% CI	*P*-value	HR	95% CI	*P*-value
TyG index	1.32	1.04–1.67	0.021	0.79	0.29–2.15	0.650
Age, years	1.00	0.99–1.01	0.838	0.99	0.97–1.01	0.184
Male	0.93	0.70–1.24	0.600	1.11	0.67–1.83	0.694
BMI, kg/m^2^	1.02	0.98–1.05	0.333			
Hypertension	1.01	0.80–1.27	0.939			
Hyperlipemia	1.17	0.91–1.48	0.217			
Current smoker	0.96	0.76–1.21	0.724			
Previous stroke	1.29	0.90–1.85	0.169			
Previous MI	1.12	0.87–1.43	0.375			
Previous PCI	1.56	1.21–2.00	0.001	1.65	0.97–2.80	0.063
Previous CABG	1.54	0.93–2.55	0.093	0.63	0.15–2.70	0.529
AMI	1.15	0.85–1.56	0.359			
LM disease	1.06	0.65–1.74	0.803			
Multivessel disease	1.61	1.21–2.14	0.001	1.55	0.87–2.76	0.134
CTO disease	2.33	1.72–3.16	<0.001	0.81	0.45–1.45	0.479
ISR disease	1.81	1.26–2.60	0.001	1.43	0.66–3.10	0.370
SYNTAX score	1.04	1.02–1.05	<0.001	1.03	1.01–1.05	0.023
Diameter of stent	0.56	0.35–0.89	0.015	0.58	0.35–0.94	0.028
Number of stents	0.98	0.86–1.11	0.719			
LVEF, (%)	0.97	0.96–0.99	<0.001	0.98	0.96–1.01	0.177
FBG, mmol/L	1.17	0.97–1.41	0.105			
HbA1c (%)	1.66	1.15–2.38	0.006	1.41	0.76–2.62	0.276
TG, mmol/L	1.14	1.04–1.26	0.005	0.98	0.57–1.69	0.953
TC, mmol/L	1.05	0.94–1.16	0.377			
HDL-C, mmol/L	0.74	0.48–1.12	0.149			
LDL-C, mmol/L	1.03	0.91–1.16	0.670			
hs-CRP, mg/L	1.03	1.00–1.06	0.079	1.02	0.97–1.08	0.401
Creatinine, mmol/L	1.00	0.99–1.01	0.821			

TyG index, triglyceride-glucose index; BMI, body mass index; MI, myocardial infarction; PCI, percutaneous coronary intervention; CABG, coronary artery bypass graft; CAD, coronary artery disease; AMI, acute myocardial infarction; LM, left main; CTO, chronic total occlusion; ISR, in-stent restenosis; SYNTAX, Synergy between PCI with TAXUS™ and cardiac surgery; LVEF, left ventricle ejection fraction; FBG, fasting blood glucose; HbA1c, hemoglobin A1c; TG, triglycerides; TC, total cholesterol; HDL-C, high-density lipoprotein cholesterol; LDL-C, low-density lipoprotein cholesterol; hs-CRP, high-sensitivity C-reactive protein.

## Discussion

The present study explored the predictive value of the TyG index in adverse cardiovascular outcomes for nondiabetic subjects who underwent PCI, and the results included the following two findings: 1) the TyG index was not related to cardiovascular adverse events, including MACCE, MACE, all-cause death, cardiac mortality, nonfatal MI, nonfatal stroke, or TVR, independently after adjusting for other cardiovascular risk factors. 2) In the LDL-C lower than 1.8 mmol/L subgroup, this index may be an independent predictive factor for MACCE.

Insulin resistance refers to the dysfunction of insulin in facilitating glucose assimilation and utilization for various reasons, leading to excessive compensatory secretion of insulin to maintain the stability of blood glucose. IR can accelerate the progression of atherosclerosis in several ways, including hyperinsulinemia and hyperglycemia, dyslipidemia (hypertriglyceridemia, low HDL-C level, and the emergence of small dense LDL), hypertension, and endothelial dysfunction (24–28). The gold standard for detecting insulin resistance is the hyperinsulinemic euglycemic glucose clamp; however, as a result of its complex operation and high cost, it is rarely carried out in clinical practice, especially in the cardiology department. HOMA-IR is another fairly accurate alternative method of assessing IR, calculated using an equation that includes fasting glucose and fasting insulin (29). However, fasting insulin is not routinely tested in the Department of Cardiology, especially for nondiabetic patients. Therefore, the application of HOMA-IR for nondiabetic patients undergoing in-hospital PCI was limited. In recent years, the TyG index, a surrogate marker, has been widely used in the clinical evaluation of IR and has been proven to be quite accurate (10, 11, 30). Additionally, the calculation of the TyG index only requires routine examination of patients in the Department of Cardiology, so it can be proverbially available in the clinic.

Many studies have shown that this indicator is associated with exacerbation and poor prognosis in CAD patients, especially those with concurrent diabetes mellitus. Wang et al. consecutively enrolled 2,531 diabetic patients who were diagnosed with ACS with a median follow-up of 36 months. They found that this index was closely related to the primary outcome, including all-cause death, nonfatal myocardial infarction, and nonfatal stroke, which can be used as a predictor (13). In a recent study, Ma et al. recruited 776 subjects with ACS combined with type 2 DM who underwent PCI. The subjects were allocated into three groups based on the percentile of the TyG index, and the median follow-up period was 30 months. The results suggested that the TyG index was positively relevant for severe cardiovascular outcomes, including all-cause mortality, nonfatal stroke, nonfatal MI, and unscheduled repeat revascularization (14). A recent nested case control study conducted by Jin and his collaborators retrospectively enrolled 1,282 CCS subjects with DM, and the participants were followed up for 36 months. The research findings indicated that the TyG level was relevant to the increased risk of composite MACCE, including cardiac mortality, myocardial infarction, postdischarge revascularization, and hospitalized unstable angina [HR (95% CI): 1.693 (1.238–2.316)] (31). However, a few studies have indicated that it is not an independent hazard factor for adverse cardiovascular outcomes in DM subjects. Vega et al. analyzed cardiovascular outcomes at a median of 14.7 years in 39,447 adult men in the Cooper Center Longitudinal Study. It can be inferred that the TyG level was not related to CAD, cardiovascular diseases (CVDs), or all-cause death (32). Most studies on the TyG index for cardiovascular adverse events have focused on patients with diabetes or ACS, while few have focused on patients without diabetes or who underwent PCI. Park et al. evaluated 16,455 subjects without DM in Korean health risk assessment study data. Over 50 months of follow-up, they found that the TyG index could predict the risk of developing ischemic heart disease (IHD) (33). A cohort study implemented by Zhao et al. enrolled 1,510 nondiabetic patients diagnosed with non-ST-segment elevation acute coronary syndrome (NSTE-ACS), and the corresponding follow-up time was 4 years. In the research process, the main endpoint was set as outcomes including all-cause mortality, MI, ischemic stroke, and repeat revascularization due to myocardial ischemia. The results indicated that this index was an independent predictive factor in NSTE-ACS patients without DM (34). However, no existing studies have concentrated on nondiabetic patients who underwent PCI. The present study included 5,489 nondiabetic Chinese participants who underwent PCI, and the baseline data showed that when the TyG level was enhanced, the younger the patients were, the higher their BMI was, and the more patients had a history of smoking and hyperlipidemia, which is basically consistent with the findings of other scholars (14–16, 35). The data suggest that younger, obese CAD patients may be more susceptible to insulin resistance. After a median 29-month follow-up, the results of the K-M curve did not show obvious differences for the primary endpoint or every secondary endpoint among the three groups. Moreover, univariate and multivariate Cox regression analyses showed that the triglyceride-glucose index was not an independent risk factor for adverse cardiovascular outcomes after adjusting for other confounding factors. Subgroup analyses showed that in the low LDL subgroup (LDL-C < 1.8 mmol/L), the TyG index was an independent hazard factor for the primary endpoint [HR (95% CI), 1.70 (1.15–2.52), *P* = 0.008, *P* for interaction = 0.045].

The results of this study are different from those of Zhao et al. for the following possible reasons. First, we selected different subjects. All patients included in the study of Zhao et al. were ACS patients, while the present study enrolled some patients diagnosed with CCS. ACS patients are prone to stress hyperglycemia and are associated with poor prognosis, which may obscure the true effect of the TyG index on prognosis (36, 37). Second, the endpoints were defined differently. The study of Zhao included ischemia-driven revascularization as the primary endpoint, while our study only included TVR. The occurrence of TVR after PCI may better reflect the poor prognosis of patients, since ischemic-driven revascularization may include nontarget vessel revascularization within a short period after discharge, which may be caused by a variety of factors, such as the willingness of doctors and patients, cost, and medical reimbursement issues. Moreover, the subgroup analyses showed that in the low LDL subgroup, this index was an independent predictive factor for MACCE. Another recent cohort study in China conducted by Zhang et al. included 1,655 nondiabetic ACS subjects whose LDL-C was below 1.8 mmol/L. The outcome suggested that the TyG index was closely related to the incidence of acute myocardial infarction, infarct size, and prevalence of revascularization (38). This may indicate that TyG, representing insulin resistance, is one of the predictors of residual cardiovascular and cerebrovascular risk in patients with well-controlled cholesterol levels when cholesterol-lowering treatment is the cornerstone of secondary prevention of CAD today. Exploring its predictive function for long-term cardiovascular prognosis in nondiabetic patients who underwent PCI remains to be further studied.

### Strengths and Limitations

This is the first study focused on the role of the TyG index in nondiabetic subjects who underwent PCI. In this research, the follow-up period was relatively long, and the corresponding sample size was sufficiently large. Fuwai Hospital is the medical center with the largest number of coronary interventional therapies in China, and the level of interventional therapy is relatively high, so the samples are representative to some extent. Of course, this study also has some limitations. For example, it was a single-center study, and the enrolled patients were all Chinese. In addition, the study did not record abdominal circumference, hip circumference, or NAFLD, which are important and independent risk factors for CVD (39, 40). Finally, the TyG index was recorded only at the time of hospitalization, and there were no follow-up data about the TyG index, which could not be used to accurately assess long-term insulin resistance levels after discharge.

### Conclusion

The TyG index was not independently relevant to adverse cardiovascular events in nondiabetic patients who underwent PCI. However, in subjects with LDL-C lower than 1.8 mmol/L, it may predict adverse cardiovascular prognosis. More large-scale prospective research should be carried out in the future to explore the predictive effect of this index in nondiabetic patients who receive PCI, especially patients with well-controlled LDL-C.

## Data Availability Statement

The datasets presented in this article are not readily available because the raw data are stored in the hospital’s computer system and are restricted from copying. Requests to access the datasets should be directed to Y-DT (drtangyida@126.com).

## Ethics Statement

The studies involving human participants were reviewed and approved by Fuwai Hospital’s Ethics Review Committee. The patients/participants provided their written informed consent to participate in this study.

## Author Contributions

JY, CS, and Y-DT participated in the study design. YZ, CL, QZ, JG, and XM participated in data collection. JY, KZ, and WW performed the statistical analysis. JY drafted the article. All authors contributed to the article and approved the submitted version.

## Funding

This work was supported by National Key R&D Program of China (2020YFC2004700) and the Beijing Nova Program (Z201100006820002) from Beijing Municipal Science & Technology Commission.

## Conflict of Interest

The authors declare that the research was conducted in the absence of any commercial or financial relationships that could be construed as a potential conflict of interest.

## Publisher’s Note

All claims expressed in this article are solely those of the authors and do not necessarily represent those of their affiliated organizations, or those of the publisher, the editors and the reviewers. Any product that may be evaluated in this article, or claim that may be made by its manufacturer, is not guaranteed or endorsed by the publisher.
